# A Resource for the Network Representation of Cell Perturbations Caused by SARS-CoV-2 Infection

**DOI:** 10.3390/genes12030450

**Published:** 2021-03-22

**Authors:** Livia Perfetto, Elisa Micarelli, Marta Iannuccelli, Prisca Lo Surdo, Giulio Giuliani, Sara Latini, Giusj Monia Pugliese, Giorgia Massacci, Simone Vumbaca, Federica Riccio, Claudia Fuoco, Serena Paoluzi, Luisa Castagnoli, Gianni Cesareni, Luana Licata, Francesca Sacco

**Affiliations:** 1 Fondazione Human Technopole, Department of Biology, Via Cristina Belgioioso, 171, 20157 Milan, Italy; livia.perfetto@fht.org (L.P.); prisca.irial@gmail.com (P.L.S.); 2 Department of Biology, University of Rome Tor Vergata, Via delle Ricerca Scientifica 1, 00133 Rome, Italy; elisamicarelli@yahoo.it (E.M.); marta.iannuccelli@gmail.com (M.I.); g.giulianigiulio@gmail.com (G.G.); saralatini207@gmail.com (S.L.); moniapugliese@gmail.com (G.M.P.); giorgiamassacci@hotmail.it (G.M.); simonevum41@gmail.com (S.V.); federicariccio1989@libero.it (F.R.); claudia.fuoco@uniroma2.it (C.F.); paoluzi@uniroma2.it (S.P.); castagnoli@uniroma2.it (L.C.); cesareni@uniroma2.it (G.C.); luana.licata@uniroma2.it (L.L.)

**Keywords:** causal network, signaling pathways, high-throughput experiments, enrichment analysis, the coronavirus disease 2019 (COVID-19)

## Abstract

The coronavirus disease 2019 (COVID-19) pandemic has caused more than 2.3 million casualties worldwide and the lack of effective treatments is a major health concern. The development of targeted drugs is held back due to a limited understanding of the molecular mechanisms underlying the perturbation of cell physiology observed after viral infection. Recently, several approaches, aimed at identifying cellular proteins that may contribute to COVID-19 pathology, have been reported. Albeit valuable, this information offers limited mechanistic insight as these efforts have produced long lists of cellular proteins, the majority of which are not annotated to any cellular pathway. We have embarked in a project aimed at bridging this mechanistic gap by developing a new bioinformatic approach to estimate the functional distance between a subset of proteins and a list of pathways. A comprehensive literature search allowed us to annotate, in the SIGNOR 2.0 resource, causal information underlying the main molecular mechanisms through which severe acute respiratory syndrome coronavirus 2 (SARS-CoV-2) and related coronaviruses affect the host–cell physiology. Next, we developed a new strategy that enabled us to link SARS-CoV-2 interacting proteins to cellular phenotypes via paths of causal relationships. Remarkably, the extensive information about inhibitors of signaling proteins annotated in SIGNOR 2.0 makes it possible to formulate new potential therapeutic strategies. The proposed approach, which is generally applicable, generated a literature-based causal network that can be used as a framework to formulate informed mechanistic hypotheses on COVID-19 etiology and pathology.

## 1. Introduction

The recent coronavirus disease 2019 (COVID-19) pandemic has motivated an unprecedented effort aimed at revealing the molecular mechanisms underlying the severe acute respiratory syndrome coronavirus 2 (SARS-CoV-2) pathology. Both low-throughput and large-scale genome/proteome-wide studies have contributed to a data flood, much of which remains to be interpreted [1,2,3,4,5]. Large-scale genome-wide studies generated different lists of genes or gene products that share a functional property, such as the ability to bind a viral protein or the feature of being up/downregulated in any given condition. Specifically, mass spectrometry (MS)-based proteomics has been applied to investigate the SARS-CoV2 dependent modulation of the proteome, phosphoproteome, and ubiquitinome [5]. Additionally, three independent studies have combined affinity purification with MS to identify host-proteins that have the potential to interact with viral proteins [3,4,5]. To fully exploit such a wealth of information, these results need to be integrated into functional maps to obtain hints about the cell functions that are perturbed by viral infection.

One popular strategy that has been developed to meet the challenge of extracting functional information for gene lists is dubbed Gene Set Enrichment Analysis (GSEA) or Over Representation Analysis (ORA) [6]. This strategy consists of a statistical approach to answer the question of whether the gene list under consideration is significantly enriched in genes that are annotated to a given pathway, or to Gene Ontology (GO) terms by experts. Fisher’s exact, chi-square, or binomial tests are commonly used to address this question [7].

A second type of approach builds on the observation that true hits from screening experiments are more connected than random genes or proteins. Accordingly, it exploits molecular interaction networks as supporting information to complement results of functional screenings. The integration of two orthogonal pieces of information has the advantage of limiting the noise that is inherent in high-throughput studies [8,9]. A comprehensive overview of pathway enrichment analysis techniques and the tools that implement them is discussed by [7]. Recently, Rubanova et al. [10] used a large integrated network of directed and undirected physical and functional relationships between proteins and siRNAs to discover functional paths leading to a phenotype of interest.

When we set out to apply these approaches to hit lists of the SARS-CoV-2 genome-wide screenings, we observed that a substantial fraction of protein hits was not annotated to pathways and as a consequence much information could not be considered.

To overcome this limitation, we first annotated cellular pathways modulated in response to SARS-CoV-2 infection in our in-house causal interactions and pathways resource, SIGNOR 2.0. Next, we developed a new network-based strategy aimed at evaluating the “functional distance” of a protein list from a set of pathways. Crucially, our strategy exploits a network of causal interactions (e.g., protein A activates/inhibits protein B), rather than physical interactions between proteins. Such networks annotate whether, in a relationship between the two connected proteins A and B, it is protein A that acts on the activity of protein B or vice versa (direction) and whether the relationship leads to the activation or inhibition of the target molecule (sign). SIGNOR 2.0 annotates approximately 26,000 experimental relationships between ~5400 proteins and is the primary resource of causal interactions with the highest coverage of the cell signaling network [11]. Our strategy enables to (i) infer cell functions that are likely to be modulated by a subset of query proteins; (ii) to suggest the molecular mechanisms underlying pathway perturbation; and (iii) estimate whether a protein list is significantly enriched for proteins involved in the regulation of key biological processes. Here we apply this novel approach to shed light on how SARS-CoV-2 interacting proteins perturb the host signaling networks, impacting crucial biological processes.

## 2. Materials and Methods

### 2.1. Curation of the SARS-CoV-2-Related Causal Interaction 

The first step of the curation process consisted in the systematic search of articles containing signaling information on the molecular mechanisms triggered by SARS-CoV-2, SARS-CoV-1, and Middle East Respiratory Syndrome (MERS) viral infections, and on their impact on cellular phenotypes. In this search we used, both standard biomedical literature search tools implemented in PubMed and Europe PubMed Central (PMC) and text mining tools, including SciBite (https://www.scibite.com/ (accessed on 25 February 2021)) and Causaly (https://get.causaly.com/covid19/ (accessed on 25 February 2021)).

The retrieved articles were further reviewed by expert curators and annotated in SIGNOR 2.0 in accordance to its curation policy. Causal interactions involving SARS-CoV-1 and MERS proteins were also incorporated in the network to make up for the lack of evidence with SARS-CoV-2.

The selected information was organized into nine cellular pathways that, according to expert reviews [12,13,14], are the most relevant to describe cellular functions that are modulated by viral infection. The cellular pathways modulated in COVID-19 disease are available in a dedicated SIGNOR 2.0 webpage (https://signor.uniroma2.it/covid/ (accessed on 25 February 2021)).

In a parallel curation effort, we aimed at integrating into the SIGNOR 2.0 causal network the 75 proteins that have been described as interactors of viral proteins, in at least two of three major proteomic experiments [3,4,5]. We succeeded in finding relevant experimental evidence for 68 of the 75 SARS-CoV-2 interacting proteins.

### 2.2. Estimating the Functional Proximity of a Protein to a Pathway

To define the functional proximity of any protein in the proteome to a pathway, we make use of the graph representation of the causal network annotated in SIGNOR 2.0. Each causal relationship in SIGNOR 2.0 is associated with a score (s) reflecting an estimate of its functional relevance. Briefly, the score in SIGNOR ranges from 0 to 1, and is calculated, integrating annotations within SIGNOR with annotations from external resources [11]. We here define the distance (d) between any two connected nodes as d = 1 − s and the length (L) of a path including more than two nodes as the sum of the distance of the edges forming the path (Figure S1A). The distance (D) between any two nodes that are not directly linked is the length of the shortest path connecting the two nodes (Figure S1B). As a causal graph is directed and signed, the distance between nodes A and B is not the same as the distance between B and A and, in addition, the distance D has a sign depending on the even or odd number of inhibitory interactions along the path.

Next, we define a global distance (GD), or proximity (P) between a query protein and a pathway, by considering the paths between the query protein and all the proteins in the pathway (Figure S1C). To this end we followed a three-step strategy:

(1) We search the cell causal interactome for paths of four steps, or less, linking the query protein and each protein in the pathway-list;
(2) We select, for each protein in the pathway, the path with the shortest distance D;
(3) If a query protein is connected to more than one protein in the pathway, we use an analogy with a parallel resistor and define the proximity P as the reciprocal of the sum of the reciprocals of the distances (D_n_) of each path linking the query protein to proteins in the pathway (Figure S1C).

As a consequence, the GD is shorter than any of the path distances as any path contributes to make the connection between the protein and the pathway tighter.

Pathways, depending on size and centrality, can connect differently to proteins in the proteome. Thus, in addition to distance, we associate to each protein pathway pair an empirical p-value estimated by calculating the distance of all the proteins in SIGNOR 2.0 from the considered pathway. Proteins that connect to the pathway with a p-value lower than a given threshold (*p*-value ≤ 0.01) are associated with the pathway.

Identification of the paths linking a query gene to any gene annotated to a pathway was programmatically implemented using the “all_simple_paths” function of the NetworkX module of the Python language [15]. This function returns all shortest paths linking any two nodes in an oriented graph. Moreover, this function allows to set a length cut-off as input parameter in order to explore only pathways with a length less or equal to the imposed cut-off. R scripting was used to run python scripts and to analyze results.

## 3. Results

### 3.1. Pathway Overrepresentation Analysis of the SARS-CoV-2 Interactome

**Figure 1:**
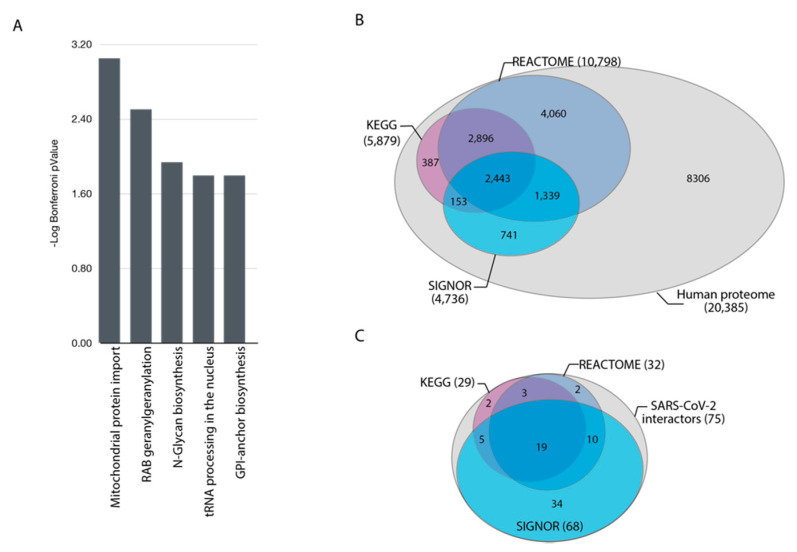
Functional analysis of severe acute respiratory syndrome coronavirus 2 (SARS-CoV-2) interactome. (**A**) ClueGO analysis of pathways enriched in the list of 75 cellular interactors of viral proteins. (**B**) Venn diagram representing the proteome coverage of the proteins annotated in the Reactome, KEGG and SIGNOR 2.0 databases. (**C**) Venn diagram illustrating the fraction of cellular partners of SARS-CoV-2 proteins ligands that are annotated in the three databases.

The COVID-19 pandemic has provoked a worldwide effort aimed at understanding the molecular mechanisms underlying viral infection. Three independent large-scale studies designed to define the SARS-CoV-2 cellular interactome [3,4,5] were recently reported. The results of these projects yielded three lists of cellular proteins that form complexes with viral proteins. The impact of these interactions on cellular functions still needs to be explained. Since the overlap between the three published datasets is surprisingly small, to limit inclusion of false positives, we here only consider 75 host proteins reported to bind SARS-CoV-2 proteins in at least two out of the three high-throughput datasets (Table S1). To shed light on the cell pathways that are likely to be modulated by these interactions, we first used pathway overrepresentation analysis. This powerful strategy relies on pathway annotation to investigate whether the elements of a gene list are significantly enriched in genes annotated to any given pathway. We checked whether this list of 75 host proteins was significantly enriched in genes annotated to pathways, using the ClueGO application [16] (Figure 1A) and identified an enrichment of genes that are involved in mitochondrial transport, tRNA processing, and maturation of proteins (e.g., RAB geranylgeranylation and N-Glycan biosynthesis) (Table S2). Although mitochondrial dysfunctions have been linked to SARS-CoV-2 pathogenesis [17,18,19,20], no additional link between proteins enriched in the list and cell functions perturbed by viral infection was revealed by this classical GSEA approach. This could be explained by noting that many proteins in the list of the 75 SARS-Cov-2 binders are not annotated to pathways (Table S2). As a matter of fact, about 40% of the human proteome is not annotated to any pathway by Reactome [21] and KEGG [22], the two pathway resources with highest proteome coverage (Figure 1B). Similarly, among the 75 SARS-CoV-2 interacting proteins, about 60% of the hits in this list are not annotated to a pathway either by KEGG or Reactome, and as such do not contribute to adding information to this analysis (Figure 1C).

We reasoned that causal relationships between proteins may be used to extend the proteome functional annotation by linking proteins of poorly characterized function to proteins annotated to pathways. Here we took advantage of SIGNOR 2.0, a database of causal information which also annotates pathways. SIGNOR 2.0 has the advantage to represent the data as a single large connected network and not as disconnected distinct pathways, whereas Reactome and KEGG are pathway resources and they show signaling interactions exclusively in the context of a pathway.

In the next section, we describe a strategy that exploits causal information to extend and expand pathway annotation to connect a larger fraction of viral interactors to a list of pathways. This is a novel network-based approach aimed at identifying pathways that are significantly “close” to a functional hit list.

### 3.2. A Causal-Network Based Strategy

**Figure 2:**
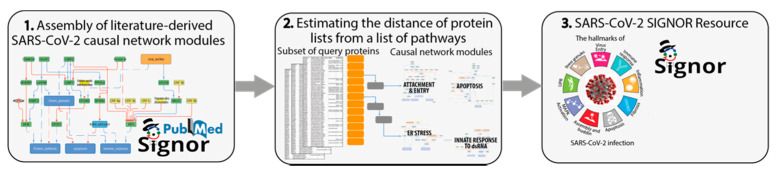
Scheme of the causal network-based strategy. The three frames enclose illustrations of the three steps used in the strategy. (1) Assembly of SARS-CoV-2 causal network and identification of functional modules. (2) Development of a graph algorithm to estimate the “functional distance” of a query list from cellular pathways that are relevant for SARS-CoV-2 infection. (3) Organize the results of this approach in a publicly available online resource.

Here, we aim to develop a generally applicable strategy to connect a subset of genes, in our case the 75 SARS-CoV-2 interacting proteins, to a list of pathways through causal interactions. To this end, we propose a three-step approach (Figure 2).

1. Identify SARS-CoV-2 modulated pathways by mining the scientific literature. Capture reports that are relevant to shed light on the biology of SARS-CoV-2 infection and organize the information in network modules (pathways), according to the affected cellular phenotypes.
2. Interpret the results of large-scale proteome-wide experiments by using graph algorithms to estimate the distance of a subset of query proteins from a list of pathways. This step enables us to evaluate which cellular functional modules are likely to be affected by each viral protein.
3. Develop a freely-accessible web resource to offer users the possibility to explore the mechanisms underlying SARS-CoV-2 infection and the supporting experimental evidence.

### 3.3. Cellular Pathways Perturbed by SARS-CoV-2 Infection: The COVID-19 Hallmark Phenotypes

**Figure 3:**
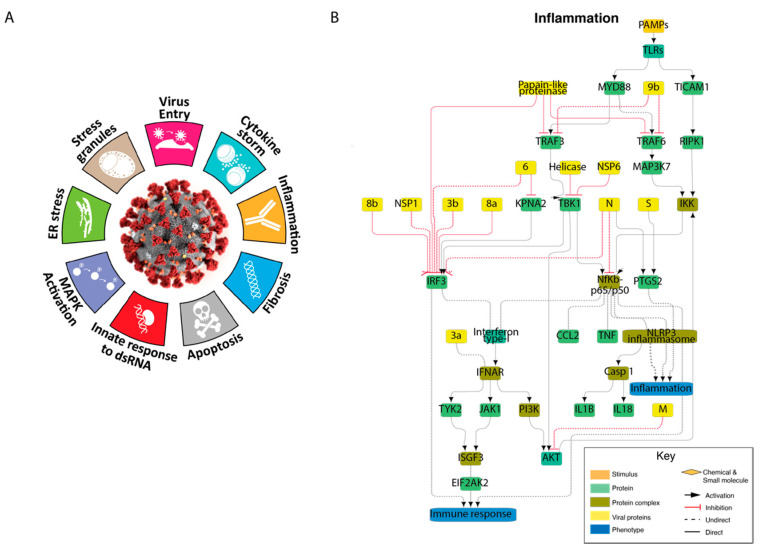
Graph representation of the SARS-CoV-2 hallmark phenotypes. (**A**) Graphical representation of coronavirus disease 2019 (COVID-19) hallmarks. (**B**) Causal network representing the modulation of the “inflammation” phenotype by viral infection; inflammation was chosen as an example. The remaining eight hallmark networks are shown in Figure S2 or can be inspected in the online resource (https://signor.uniroma2.it/covid/ (accessed on 25 February 2021)). Cellular and viral proteins are represented as green and yellow rectangles, respectively. Protein complexes are in a different tone of green whereas large blue rectangles label phenotypes. Chemicals targeting important nodes are represented as orange rhombi. Black and red arrows represent activations or inhibitions. Indirect relationships are drawn with dashed lines.

As a first step, we set out to organize the experimental evidence on the functional perturbations of cellular mechanisms caused by SARS-CoV-2 infection. We mined the scientific literature to capture relevant reports to shed light on the biology of SARS infection. As described in the method section, the captured information was reviewed by expert curators, annotated in the SIGNOR 2.0 database [11] and organized into nine network-modules representing the impact of viral proteins on cellular phenotypes (Figure 3 and Figure S2). These nine networks represent the cellular functions perturbed by SARS-CoV-2 as described by expert reviews [12,13,14]. Here, we dubbed these phenotypes SARS-CoV2 hallmarks in analogy with the cancer hallmarks of Hanahan and Weinberg [23]. The nine modules can be combined in a single network representing our current understanding of the molecular mechanisms underlying SARS-CoV-2 pathology (see online resource (https://signor.uniroma2.it/covid/ (accessed on 25 February 2021)). Importantly, the graph representations also include chemicals that target critical nodes of the network, thereby offering clues about strategies to rewire pathway activities.

The experimental evidence underlying each of the hallmark graphs can be inspected and the graphs downloaded from the online resource (https://signor.uniroma2.it/covid/ (accessed on 25 February 2021)).

### 3.4. Defining the Functional Distance of a Query Protein from a Pathway

We next developed an algorithm that measures the graph distance of a protein from a pathway. This is based on the consideration that “pathways” are not isolated entities with defined boundaries, but rather they are intimately embedded in an intricate web of connections that forms the cell network. In this perspective, any node connected in the network can affect the activity of a pathway via a set of causal relationships forming a path from the protein to the pathway. The algorithm allowed us to “walk” across the cell network to find the shortest path of causal interactions linking any query protein to a pathway. In this way it is possible to expand the annotated pathways to include proteins that are significantly closer to the pathway than expected, on a random basis. Our approach is based on the human causal network annotated in SIGNOR 2.0, which, importantly, assigns a reliability score to each interaction depending on supporting experimental evidence and functional relevance. This score can be used to annotate each edge in the graph with a distance estimating the functional closeness between the two partner proteins.

The strategy (Figure S1), as described in the method section, allows us to estimate for each protein in the proteome its “functional distance” from a pathway.

We need to consider, however, that different pathways depending on size and “centrality” are more or less reachable by any protein in the proteome. Many proteins in the proteome network would need fewer steps to reach a large and central pathway rather than a smaller and peripheral one. To take this into account, once we define the distance of a protein from a pathway, we estimate the probability that this distance or smaller occurs by chance by calculating the distance distribution of all the proteins in the SIGNOR 2.0 network. This allows us to empirically estimate the p-value associated with the observed distance. We consider this p-value as a measure of the proximity of the query protein to the pathway. Proteins with a *p*-value smaller than 0.01 are considered closely associated with the pathway. In essence, this approach uses causal relationships within the cell network to extend pathway annotations to proteins that are functionally linked to the pathway.

### 3.5. Genes Modulating Autophagy: A Test Case

As a test for our strategy, we used a list of genes observed to modulate autophagy in a genome-wide CRISPR knock-out screening [24]. Specifically, we considered the top 100 genes up (50) or downregulating (50) autophagy (Table S3). We next applied our previously developed strategy to ask whether any of the non-annotated genes in this list is significantly closer to the autophagy pathway or other pathways related to the autophagic process. As target pathways we used all the pathways annotated in SIGNOR 2.0 plus the list of SARS CoV-2 hallmark pathways in Figure 3A. 

By applying the strategy outlined above, we observed that 14 out of the 100 genes in the list are significantly closer, than expected on a random basis (*p*-value < 0.01), to the autophagy pathway and six to the lysosome pathway (Table S4). This answers the question of “which genes in a list are likely to modulate the autophagy function” and provides a way to prioritize genes for more detailed studies.

A second question is “whether the list of 100 putative modulators of autophagy is significantly enriched for genes that can be linked to the autophagy pathway” by our approach. By applying a Fisher test, we conclude that the 14 genes linked to autophagy in the experimental list are significantly more numerous than expected on a random basis (*p*-value ≤ 10^−11^). A similar result could be obtained by applying standard enrichment tools such as ClueGO (Table S5). However, our approach allowed us to identify how non annotated genes could modulate autophagy. Inference that could not be reached by applying ClueGO. In addition, it also offers a hypothesis about the causal path linking the query protein to the function.

### 3.6. Exploring the Function of SARS-CoV2 Interacting Proteins

Next, we focused on the 75 cellular proteins observed to bind SARS-CoV-2 proteins in at least two out of the three recently published datasets [3,4,5]. By applying our strategy, we identified the shortest paths of these SARS-CoV-2 interacting partners to the same subset of pathways used for the autophagy test case.

**Figure 4:**
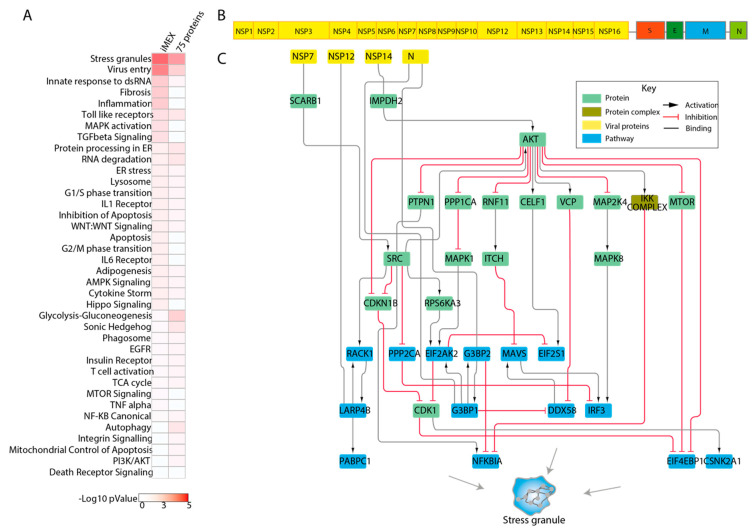
Inferred causal paths linking viral proteins to cellular pathways. (**A**) Heatmap illustrating the −Log(*p*-values) of pathway proximity enrichment of the two interactor lists considered in our approach. The right and left columns report in a red to white color scale the −Log10 *p*-value of the enriched pathways identified in the list of the 75 protein hits and the 116 protein interactors curated by the IMEx consortium respectively. (**B**) Schematic illustration of the SARS-CoV-2 genome. (**C**) Graph representation of the inferred functional paths underlying the modulation of stress granule formation in human host cells by SARS-CoV-2 interactors. Viral proteins are in yellow. The proteins that are annotated to the “stress granule” pathway are in blue. In green are the human proteins that bridge the viral proteins to the proteins annotated to the stress granule pathway.

By applying our pathway proximity approach, we observed that the list was significantly enriched in proteins that may participate in the regulation of stress granule formation, a process that has been suggested to be modulated by viral infection [24,25]. Some of the results with highest significance were discussed in previous reports. An example is represented by the nucleocapsid protein (N) targeting, G3BP1 and G3BP2, which disassemble stress granules and facilitate viral production [26]. In addition, the interactor list is also enriched for proteins that are functionally close to the process of viral entry and to proteins involved in glycolysis and gluconeogenesis. SARS-CoV-2 was shown to modulate the metabolism of infected cells that become highly glycolytic, thereby facilitating viral replication [27]. The paths linking viral proteins to the process of viral entry implicate IMPDH2 in the viral entry pathway. Consistently, IMPDH inhibitors have been shown to interfere with coronavirus replication in Vero E6 cells [28,29]. Likewise, PIK3C3 was found to be crossed over by paths connecting viral proteins to processes of virus entry, ER stress, and innate response to dsRNA and stress granules. PIK3C3 it was shown to be involved in the initial phase of the viral life cycle, such as endocytosis, fusion, translation, and replication [30], and use of PIK3C3 inhibitors have been shown to have antiviral effects [31,32]. We also applied the same approach to a larger list of SARS-CoV-2 interacting proteins (116), recently curated by the IMEx consortium [33]. This list, in addition to the results of the high throughput approaches, also includes results of low throughput experiments that have been supported by more than one report. In addition to “stress granules” and “virus entry”, this list was found to be significantly enriched in proteins that are functionally closer to innate response to dsRNA, fibrosis, and inflammation (Figure 4A).

Importantly, our approach also enabled us to infer the functional path connecting SARS-CoV-2 proteins to crucial phenotypes underlying COVID-19 pathogenesis. As shown in Figure 4B,C, viral proteins NSP14, NSP12, NSP7, and protein N are inferred to perturb the stress granule pathway via the AKT hub, which in turn activates different kinases, including IKK, MAPK, and mTOR.

We believe that this newly developed strategy could be useful to interpret the results of any high throughput approach to SARS-CoV-2 pathogenesis, and are offering an online resource (https://signor.uniroma2.it/covid/ (accessed on 25 February 2021)) where, after reviewing the currently available information on SARS-CoV-2, we display it in a network format. By clicking the nodes and edges of the graphs representing the cell pathways involved in COVID-19 pathogenesis, it is possible to connect to the literature supporting the interactions between the viral and cell proteins whose functions have an impact on relevant cell phenotypes.

In addition, users looking for suggestions about the possible paths leading from a list of proteins to SARS-CoV-2 hallmark phenotypes can query the resource with user defined lists of proteins. The system returns information about the pathways that are enriched in the list together with the causal path connecting the proteins in the list and hallmark phenotypes.

## 4. Discussion

The work presented here was motivated by the ambition to organize the information on SARS-CoV-2 biology into a functional framework. By incorporating much of the recently published findings, we offer a useful resource to support experiment design and result interpretation. Although our understanding of SARS-CoV-2 biology is presently largely incomplete [3,4,5], it is hoped that this work could also provide a useful scaffold to help identifying new targets and devise novel therapeutic strategies.

We have made an effort to screen the literature, and archives, looking for manuscripts reporting evidence that could link viral proteins to the host causal protein network. This information was integrated into the SIGNOR database and organized into simpler, more compact, functional modules. The resulting models are not conclusive static-snapshots of SARS-CoV-2 biology and their predictions should be challenged by confronting them with the results of ongoing projects aimed at revealing the molecular mechanisms underlying SARS-CoV-2 biology. This work has been done in synergy with the COVID-19 Disease Map project, a broad community-driven effort to build a knowledge repository of molecular mechanisms of COVID-19 [34].

Our work, in addition to applying a network-based approach to organize the information on COVID-19 pathology, was also aimed at deriving information from protein lists generated by genome-wide functional screenings. A common strategy for this task is GSEA, which relies on annotation of pathway databases to investigate whether the query protein list is enriched for proteins annotated to a pathway [6]. To this end, many resources have endeavored to compile lists of genes that are associated with host pathways. However, defining pathways and deciding the genes/proteins that play a role in that signaling cascade is an ill-defined task as demonstrated by the observation that pathway lists defined by different resources have limited overlap [35]. This is because pathways are useful mental abstractions whose functional boundaries are not clearly defined. Thus, two experts may have different opinions on whether any specific gene belongs to a pathway or not. To overcome this limitation, we developed a new strategy that makes use of causal information annotated in the SIGNOR 2.0 database [11] to estimate the “functional distance” of any protein in the proteome from a pathway. By this approach we extend pathway annotations to all the proteins in the cell causal network. 

Our strategy is general and can be applied to hit lists from any functional screening. Importantly, the proposed approach is independent from the expert decision of assigning a protein to a pathway or not. Once one chooses some key pathway-proteins, all the remaining proteins in the cell causal network can be assigned a score that estimates its functional proximity to the pathway. Thus, our strategy makes it possible to identify a larger number of proteins whose activity may modulate a pathway as compared with standard GSEA methods. 

## 5. Conclusions

We applied this new approach to lists of proteins that were found to interact physically with SARS-CoV-2 viral proteins. We identified possible paths linking viral interactors to cellular pathways. For instance, our analysis led us to conclude that NSP7, NSP12, NSP14, and N viral proteins have the potential to modulate the pathway of stress granule formation via activation of AKT. It is important to stress that, although these paths are assembled starting from experimentally supported causal protein relationships, the suggested pathways have not been validated in the specific context and, as such, some of them may turn out to be functionally irrelevant. One additional limitation of our approach is that the fraction of proteins that is (or can be) incorporated into the cell causal network is still limited because of insufficient experimental evidence or incomplete curation coverage of the literature. We want to emphasize here that the ranking of the paths that are proposed to link viral proteins to pathways are influenced by the strategy used by the SIGNOR database to assign a score to each causal edge. This is currently under revision. Additionally, we observed that the shortest paths tend to go across nodes with high degrees. To give a higher chance to a large variety of paths we are planning to revise the algorithm to penalize paths that use hubs to reach cellular pathways.

Finally, here we present a novel generally applicable strategy, relying on literature-based causal networks that can be used as a framework to improve our understanding of COVID-19 pathology and to identify new therapeutic targets. 

## Supplementary Materials

The following are available online at https://www.mdpi.com/2073-4425/12/3/450/s1, Figure S1: Illustration of the main steps in defining the pathway distance of a query protein, Figure S2: Graph representation of the nine SARS-CoV-2 hallmark phenotypes. Table S1. List of the 75 SARS-CoV-2 interacting proteins. Table S2. Clue-GO enrichment analysis of the 75 SARS-CoV-2 interacting proteins. Table S3. List of the top 100 gene hits controlling autophagy. Table S4. Paths from the autophagy-related genes to SIGNOR 2.0 pathways. Only significant (*p*-value < 0.01) paths have been shown. Table S5. Clue-GO enrichment analysis of the 100 gene hits regulating autophagy.
