# Supplementary material for: A Resource for the Network Representation of Cell Perturbations Caused by SARS-CoV-2 Infection
Source: Genes (Basel). 2021 Mar 22;12(3):450. doi: 10.3390/genes12030450 (PMC8004236; doi:10.3390/genes12030450)
Supplement: Supplementary file 1 [file genes-12-00450-s001.zip › Supplemetary figures and tables/Supplementary figures.docx]

**
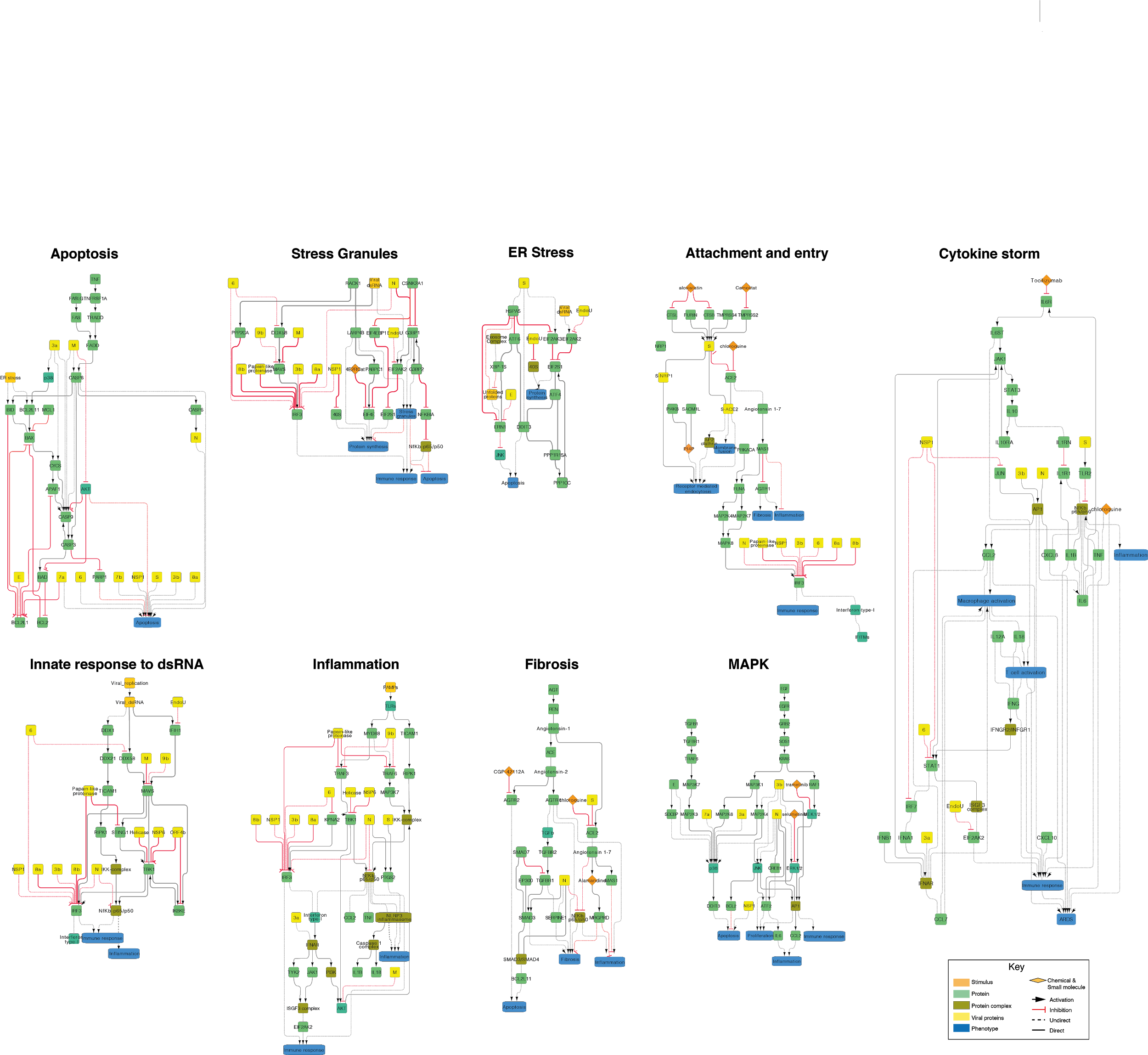
**

**Figure S1. Graph representation of the nine SARS-Cov-2 hallmark phenotypes.** Cellular and viral proteins are represented as green and yellow rectangles respectively. Protein complexes are in a different tone of green while large blue rectangles label phenotypes. Chemicals targeting important nodes are represented as orange rhombi. Black and red arrows represent activations or inhibitions. Indirect relationships are drawn with dashed lines


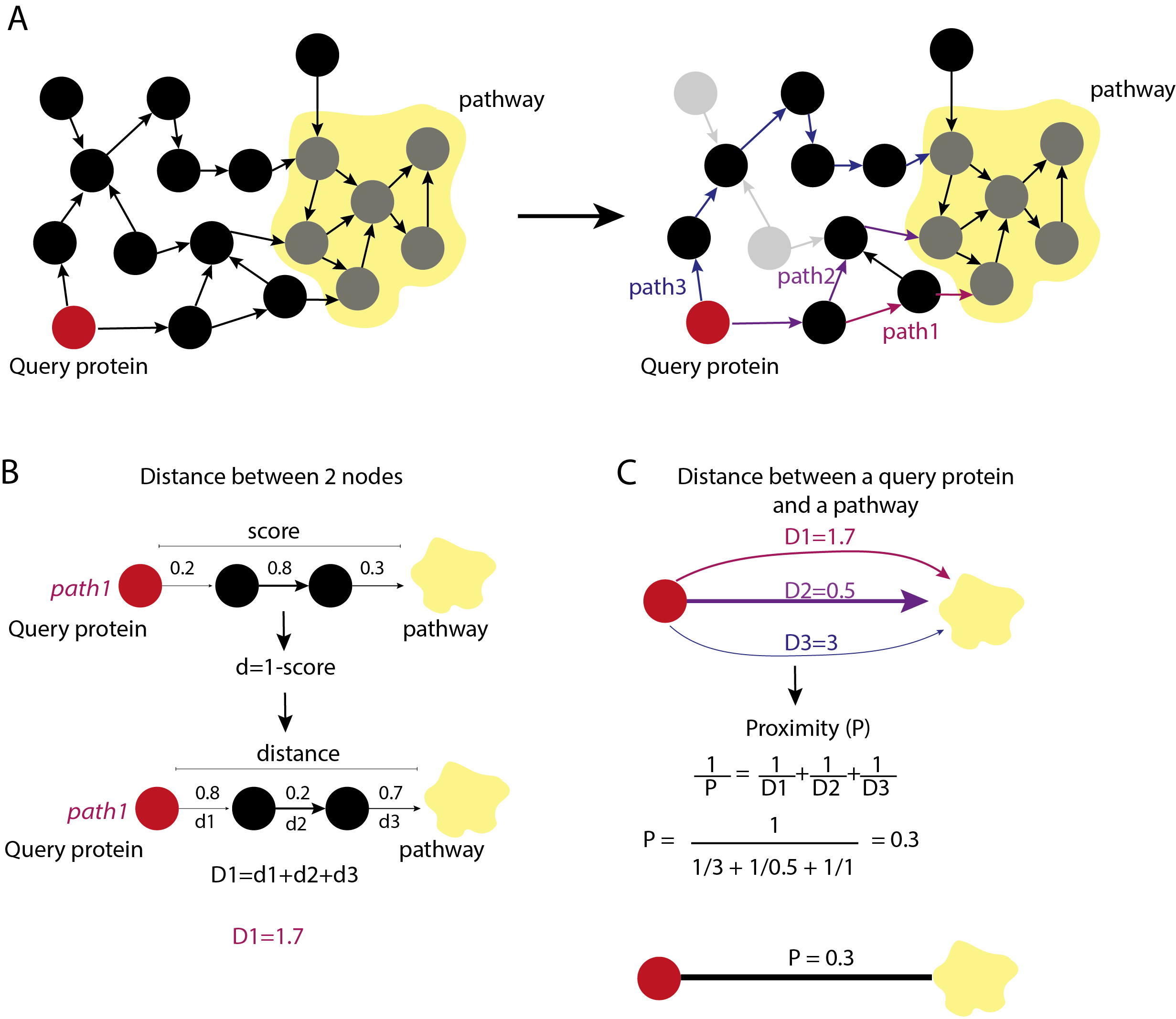


**Figure S2.  Illustration of the main steps in defining the pathway distance of a query protein.** **A**) The aim is that of assigning a graph “distance” of a query protein (red) from a list of proteins (pathway, grey nodes) in a weighted graph (left). All the possible paths leading from the query protein to the protein pathway (yellow shade) are listed. **B**) As the graph is weighted with a reliability score it is possible to assign to each edge a distance defined as 1-score. The path length is computed as the sum of the distances of the edges forming the path. The distance (D) between any two proteins in the graph is defined as the length of the shortest path between the two proteins. **C**) We next define the proximity of a query protein to a pathway by combining all the shortest paths between the query protein and the nodes annotated to the pathway. The different path lengths are combined by taking the inverse of the sum of the inverses of the different path lenghts.
